# Remote and unsupervised digital memory assessments can reliably detect cognitive impairment in Alzheimer's disease

**DOI:** 10.1002/alz.13919

**Published:** 2024-06-12

**Authors:** David Berron, Emil Olsson, Felix Andersson, Shorena Janelidze, Pontus Tideman, Emrah Düzel, Sebastian Palmqvist, Erik Stomrud, Oskar Hansson

**Affiliations:** ^1^ Clinical Memory Research Unit, Department of Clinical Sciences Malmö Lund University Lund Sweden; ^2^ German Center for Neurodegenerative Diseases Magdeburg Germany; ^3^ Memory Clinic Skåne University Hospital Malmö Sweden; ^4^ Institute for Cognitive Neurology and Dementia Research Otto‐von‐Guericke University Magdeburg Germany; ^5^ Institute of Cognitive Neuroscience University College London London UK

**Keywords:** Alzheimer's disease, ambulatory assessments, blood‐based biomarkers, digital cognitive markers, ecological momentary assessments, memory, mHealth, plasma marker, smartphone‐based unsupervised assessments

## Abstract

**INTRODUCTION:**

Remote unsupervised cognitive assessments have the potential to complement and facilitate cognitive assessment in clinical and research settings.

**METHODS:**

Here, we evaluate the usability, validity, and reliability of unsupervised remote memory assessments via mobile devices in individuals without dementia from the Swedish BioFINDER‐2 study and explore their prognostic utility regarding future cognitive decline.

**RESULTS:**

Usability was rated positively; remote memory assessments showed good construct validity with traditional neuropsychological assessments and were significantly associated with tau‐positron emission tomography and downstream magnetic resonance imaging measures. Memory performance at baseline was associated with future cognitive decline and prediction of future cognitive decline was further improved by combining remote digital memory assessments with plasma p‐tau217. Finally, retest reliability was moderate for a single assessment and good for an aggregate of two sessions.

**DISCUSSION:**

Our results demonstrate that unsupervised digital memory assessments might be used for diagnosis and prognosis in Alzheimer's disease, potentially in combination with plasma biomarkers.

**Highlights:**

- Remote and unsupervised digital memory assessments are feasible in older adults and individuals in early stages of Alzheimer's disease.
- Digital memory assessments are associated with neuropsychological in‐clinic assessments, tau‐positron emission tomography and magnetic resonance imaging measures.
- Combination of digital memory assessments with plasma p‐tau217 holds promise for prognosis of future cognitive decline.
- Future validation in further independent, larger, and more diverse cohorts is needed to inform clinical implementation.

## 1 BACKGROUND

While there has been significant progress in fluid and neuroimaging biomarkers to detect pathological changes in Alzheimer's disease (AD), most cognitive measures that are used in health care and clinical trials were initially designed to detect overt cognitive impairment and novel developments still lag behind.^1^, ^2^ This is in stark contrast to recent discoveries on the functional architecture of episodic memory and its relationship to the spatial progression patterns of AD pathology.^3^, ^4^ Recent work on the functional neuroanatomy of episodic memory has highlighted memory networks in the medial temporal lobe (MTL) and the neocortex that are involved in specific memory functions and are affected in different stages of AD.^4^, ^5^, ^6^, ^7^ Episodic memory requires pattern separation and completion processes that are primarily mediated by MTL subregions. While the dentate gyrus is involved in pattern separation^8^, ^9^ and reduces memory interference between similar events, the hippocampal Cornu Ammonis 3 mediates pattern completion processes in interplay with neocortical regions.^10^ Within the hippocampal entorhinal circuitry there exist partly segregated pathways where object information is primarily provided from the perirhinal cortex via the anterior‐lateral entorhinal cortex. Spatial information via the parahippocampal cortex is transferred additionally through the posterior parts of the entorhinal cortex.^3^, ^11^, ^12^, ^13^, ^14^, ^15^ Taken together, there is converging evidence that short‐term mnemonic discrimination of object and scene representations, in addition to long‐term memory, is impaired in the predementia stages of AD.^16^

Traditional neuropsychological assessment suffers from significant limitations such as high participant burden and impracticality of implementing test formats such as frequent test repetitions or long‐term delay formats. Thus, traditional neuropsychological assessments become increasingly difficult for clinical trials in preclinical and early‐stage symptomatic AD populations which are gradually implementing decentralized clinical trial structures for case‐finding and monitoring,^17^ for example, in TRAILBLAZER‐ALZ 3 (ClinicalTrials.gov Identifier: NCT05026866). In this context, unsupervised and remote digital cognitive assessments via smartphones and tablets offer a promising avenue to improve case‐finding, monitoring, and prognosis in both clinical trial and health‐care settings.^18^ Approaches from several groups, including our own, have recently demonstrated that remote and unsupervised assessments are feasible in healthy older adult populations and those at risk of AD.^19^, ^20^, ^21^, ^22^, ^23^, ^24^ Furthermore, this work showed that remote and unsupervised assessments can support the identification of mild cognitive impairment (MCI) patients in a memory clinic setting^25^ and potentially even β‐amyloid (Aβ) positive but cognitively unimpaired (CU) participants.^19^, ^26^, ^27^ The aim of the present study was to evaluate the feasibility and usability of unsupervised and remote digital memory assessments, their construct validity in reference to traditional neuropsychological assessments, their retest reliability, and their relationship with fluid and imaging biomarkers of AD. To that end, we implemented two non‐verbal visual memory tasks based on recent insights into the functional anatomy of episodic memory, available on the neotiv digital platform (https://www.neotiv.com/en),^21^, ^24^ in a subset of the Swedish BioFINDER‐2 study.

## 2 METHODS

### 2.1 Recruitment into smartphone‐based add‐on study

A total of 187 individuals gave written informed consent to participate in the 7 Tesla magnetic resonance imaging (MRI) study arm of the Swedish BioFINDER2 study and were offered participation in biweekly smartphone‐based remote cognitive assessments across a 12‐month period. Of those offered participation, 160 individuals agreed to complete the smartphone‐based assessments. A brief pen‐and‐paper questionnaire about experience with smartphones and mobile apps was completed and most participants downloaded and installed the neotiv app directly after the MRI session on their own mobile device and completed the first phase of the first digital memory assessment session on site. A comprehensive user manual was distributed which included download and installation instructions, and those individuals who did not download and install the app on‐site successfully completed it without supervision from their home. The study was approved by the Regional Ethics Committee in Lund and the Swedish Ethical Review Authority.[Fig alz13919-fig-0001]

Following completion of the initial app‐hosted cognitive assessment, participants were notified to complete memory assessments every two weeks for 12 months but could also be postponed. Each of the assessments consisted of a two‐phase session. The two phases were either two halves of a mnemonic discrimination task (MDT‐OS) or encoding and retrieval phases of an object‐in‐room recall (ORR) task (see details of the tasks below). Each phase took less than 10 minutes. Thus, overall participants could complete up to 12 sessions for each memory task within a period of approximately 12 months. To minimize potential practice effects from repeated testing, 12 independent difficulty‐matched parallel test versions for each memory task were used.^24^ After each task completion, participants indicated their subjective task performance and their concentration level on a 5‐point scale (1 = very bad, 2 = bad, 3 = moderate, 4 = good and 5 = very good), and whether they had been distracted during the task (yes/no). In the current cross‐sectional analyses, the first valid session of each participant was used.

Push notifications were used to notify individuals about available tasks and to remind them daily for five consecutive days if a task had not been initiated. At the beginning of each test session, participants were asked to go to a quiet environment, wear their glasses if needed and to adjust their screen's brightness to see the pictures clearly. They also received a short practice session for the initial test session as well as for all future sessions.

**FIGURE 1 alz13919-fig-0001:**
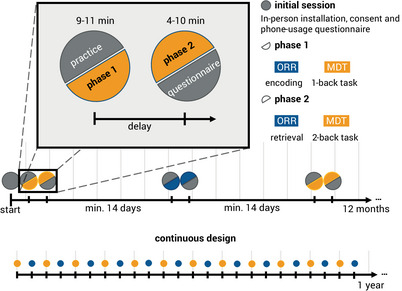
Timeline of the study protocol. Participants enlisted for a 12‐month study of biweekly remote and unsupervised memory assessments of the MDT‐OS and ORR. In the initial in‐clinic session, they gave consent, got a brief introduction, answered a short questionnaire on their phone usage, and completed the first task. Every 2nd week, they received a short training session, followed by phase 1 of their respective task: encoding for ORR, and 1‐back task for MDT‐OS. After finishing phase 1, they were notified when the next phase was available, and could perform it straightaway or postpone if inconvenient; that is, there was a minimum delay of 24 h for the MDT‐OS and a minimum delay of 60 minutes for the ORR, but it was often extended by the participants. Phase 2 consisted of retrieval for ORR, and 2‐back task for MDT‐OS. It was followed by ratings regarding concentration, distraction throughout and subjective difficulty of the task. MDT‐OS; Mnemonic Discrimination Task for Objects and Scenes; ORR, objects‐in‐room‐recall task.

At the end of the study, 38 participants completed telephone interviews to provide feedback on usability and user experience throughout the study. The full study timeline can be seen in Figure 1.

**RESEARCH IN CONTEXT**

1. **Systematic review**: We reviewed the literature on remote digital cognitive assessments in Alzheimer's disease (AD) using traditional sources (e.g., PubMed) and focused on comparable remote digital cognitive assessments to our own. We included work on brief, while not remote and unsupervised, assessments that have been used in combination with novel plasma markers for prognosis of disease progression.
2. **Interpretation**: Our findings show that unsupervised remote digital memory assessments are feasible in older adults and early AD populations, that they are associated with traditional in‐clinic cognitive assessments and imaging biomarkers and that they have moderate to good retest reliability. In addition, our findings demonstrate that a combination of plasma and digital cognitive markers can significantly contribute to the prediction of future cognitive decline.
3. **Future directions**: Replication of our results in further independent, larger, and more diverse cohorts will be necessary in future studies.

### 2.2 Smartphone‐based memory assessments

#### 2.2.1 Mnemonic discrimination of objects and scenes

**FIGURE 2:**
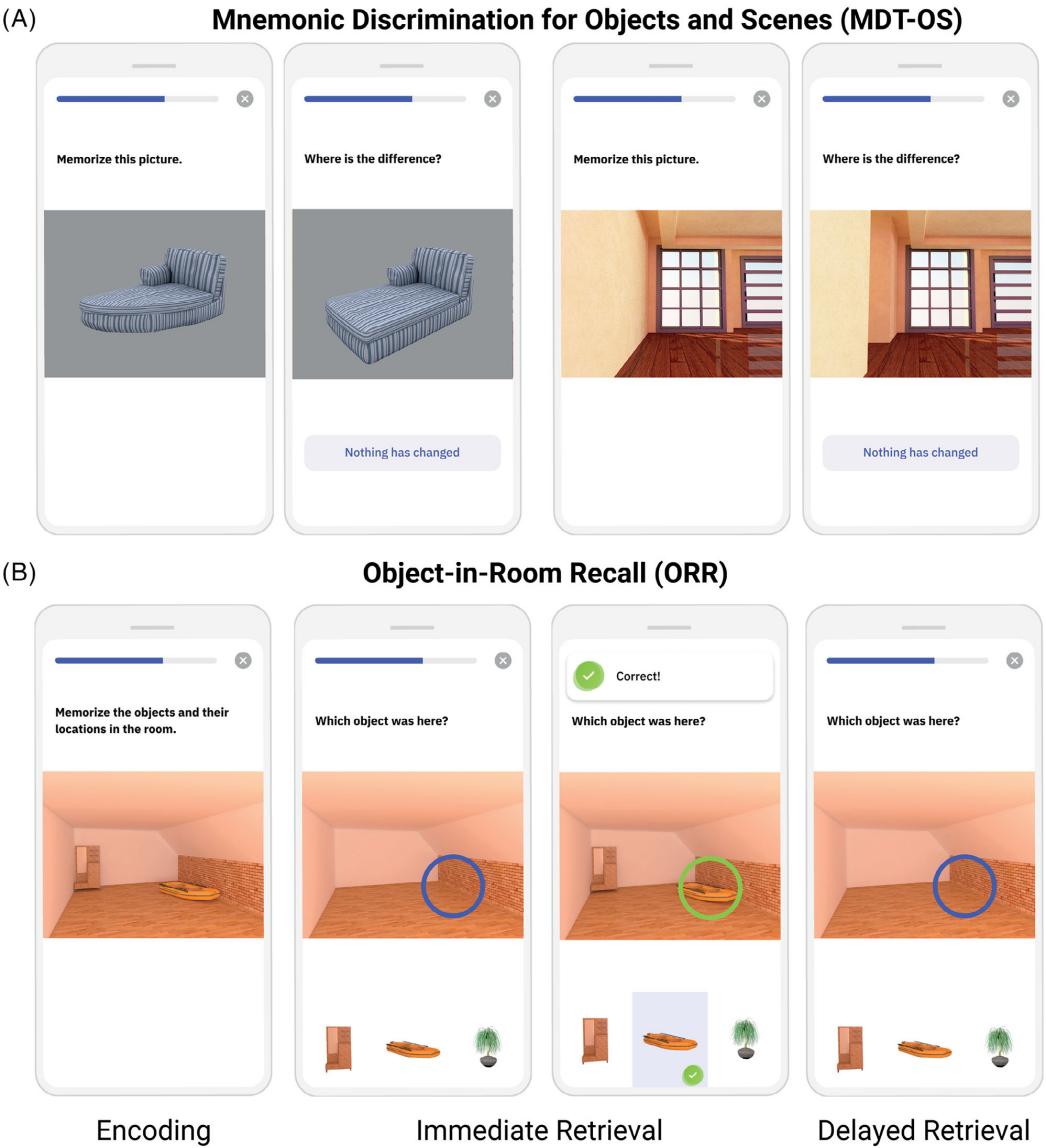
Smartphone‐based memory tasks. (A) MDT‐OS and (B) ORR test. MDT‐OS; Mnemonic Discrimination Test for Objects and Scenes; ORR, Objects‐In‐Room Recall.

In this continuous recognition task, individuals were presented with computer‐generated images of various indoor objects and empty rooms, which were either exactly repeated, or slightly altered (see Figure 2A). Participants had to indicate whether an image was an identical repetition of a previous image (tap on a button), or had been modified (tap on the location of change). One session consisted of 64 image pairs (32 object pairs, 32 scene pairs), half of which were modified, and half of which were repeated. To optimize engagement while minimizing the subjective burden of participating, each session was split into two phases and automatically scheduled on two consecutive days with a 24‐h delay between phases. The first phase was presented as a one‐back task while the second phase was presented as a two‐back task. The Mnemonic Discrimination of Objects and Scenes (MDT‐OS) has been designed to tax hippocampal pattern separation; a memory mechanism needed to discriminate between similar memories. Earlier studies using functional MRI have shown that especially subregions in the human MTL are involved in this task.^3^, ^11^, ^13^ The test provides a hit rate, a false alarm rate and a corrected hit rate (hit rate minus false alarm rate) for both the object and scene condition. The averaged corrected hit rate across the scene and the object condition was used as the main outcome measure in the following analyses and object, and scene‐specific corrected hit rates were additionally investigated.

#### 2.2.2 Objects‐In‐Room Recall

In this task, participants had to memorize a spatial arrangement of 2 objects within a room. Following the encoding phase, a blue circle highlighted the previous position of one of the objects in the same but now empty room and the participant had to identify the correct object from a selection of three in an immediate retrieval phase (see Figure 2B). Among the 3 possible objects, 1 was the correct one that was previously shown in the now highlighted position (target), another belonged to the same room but had previously been shown at a different position (correct source distractor), and the third had previously been shown in a different room (incorrect source distractor). They learned 25 such object‐scene associations. After 60 minutes, the participant was notified via push notification to complete an identical but delayed retrieval phase with a randomized stimulus order. The ORR has been designed to tax hippocampal pattern completion, a memory mechanism needed to restore full memories from partial cues.^10^, ^28^ In the test, the assessment of recall is graded and allows to separate correct episodic recall from incorrect source memory. Thus, correct recall excludes the choice of an object that was present in the same room but at a different location (wrong source memory for specific location), and an object that was not present in the room but nevertheless associated with another room during encoding (wrong source memory for overall location). The test provides an immediate recall (ORR‐IR; 0–25) and a (ORR‐DR [delayed recall]; 0–25) score. Here, we use the ORR‐DR.

#### 2.2.3 Quality control procedures

In the current analyses, we analyzed data collected up until the data release in July 2023. Recruitment of participants from the Swedish BioFINDER‐2 study was done between February 2019 and February 2022. Only participants who had completed at least 1 full session, that is, both phases of a task, were included. Regarding the very first assessment of MDT‐OS and ORR‐DR, 6% of test sessions exceeded the threshold for missing responses (maximum of 25% of missing responses per session), and 17% of test sessions exceeded the maximum length of the delay period (> 240 minutes) before filtering. These sessions were excluded during quality assessment. Excluded test sessions were replaced by valid subsequent sessions where possible. As a result, 81% of test sessions we report here were from the first 2 MDT‐OS and ORR‐DR sessions respectively while 19% were from subsequent test sessions (mean test session = 2.1, range = 1–11).

### 2.3 Cerebrospinal fluid and plasma analysis

Cerebrospinal fluid (CSF) Aβ42 and Aβ40 concentrations were measured using the Elecsys assays (Roche Diagnostics). Participants were stratified for Aβ status using the CSF Aβ42/40 ratio with a predefined cut‐off of 0.08.^29^ Plasma levels of p‐tau217 were quantified with the MesoScale Discovery‐based immunoassay developed by Lilly Research Laboratories as previously described.^30^

### 2.4 Imaging acquisition

#### 2.4.1 MRI

T1‐weighted images were acquired on a 3T Siemens Prisma scanner (Siemens Medical Solutions, Erlangen, Germany) with a 64‐channel head coil using an MPRAGE sequence (in‐plane resolution = 1 × 1 mm^2^, slice thickness = 1 mm, TR = 1900 ms, echo time = 2.54 ms, flip‐angle = 9°). The 7T data acquired in the project were not analyzed for this manuscript.

#### 2.4.2 Tau and Aβ‐PET

All study participants underwent positron emission tomography (PET) scans on a digital GE Discovery MI scanner (General Electric Medical Systems). Approval for PET imaging was obtained from the Swedish Medical Products Agency. For tau‐PET imaging, the participants were injected with 365 ± 20 MBq of [18F]RO948, and LIST mode emission data was acquired for each scan 70–90 minutes ([18F]RO948) post injection. Aβ‐PET imaging was performed on the same platform 90–110 minutes after the injection of ∼185 MBq [^18^F]flutemetamol.^31^

### 2.5 Imaging analysis

#### 2.5.1 ROI segmentation and estimates of volume and thickness

Individual volume of the anterior and posterior hippocampus and median thickness of area 35 were defined on T1‐weighted images (1 × 1 × 1 mm^3^ resolution) using Automatic Segmentation of Hippocampal Subfields‐T1 (Xie et al., 2019) and a multi‐template thickness analysis pipeline.^32^ All subregional masks were visually assessed.

#### 2.5.2 Standardized uptake value ratio measures

*[^18^F]RO948*: Standardized uptake value ratio (SUVr) images were calculated for area 35 using an inferior cerebellar reference region.^33^ Partial volume correction was performed using the geometric transfer matrix method^34^ extended to voxel‐level using region‐based voxel‐wise correction.^35^ To reduce the influence of off‐target binding, choroid plexus tau‐PET signal was regressed from hippocampal measures. *[^18^F]Flutemetamol*: A cortical composite SUVr as well as a measure of early amyloid deposition in the precuneus was calculated using the whole cerebellum as reference region.^36^, [Table alz13919-tbl-0001]

#### 2.5.3 Traditional cognitive measures

As a measure representing episodic memory, we used the delayed 10‐word list recall test from the Alzheimer's Disease Assessment Scale—Cognitive Subscale (Rosen et al., 1984). The learning trial of the 10 words was repeated 3 times. After a distraction task (Boston Naming—15 items short version^37^), the participant was asked to freely recall the 10 words (“delayed recall”). Delayed recall was scored as number of errors (i.e., 10 minus correct recalled words), so that a higher score entailed worse memory performance. For global cognition, we used the Mini‐Mental State Examination (MMSE^38^; scale from 0–30). For attentional and executive function, we used the Symbol Digit Modalities Test (SDMT,^39^ 1 point for every correct answer within the response time of 90 seconds) and verbal fluency (Animal fluency; number of correct animals within the response time of 60 seconds). In addition, we used the corrected hit rates for object and scene memory derived from an on‐site version of the test that was completed during a functional MRI scan. In the end, we calculated a composite score similar to the Preclinical Alzheimer Cognitive Composite 5 (PACC5^40^), the modified PACC (mPACC), using the average of the z‐standardized scores of Alzheimer's Disease Assessment Scale (ADAS) delayed recall (counted twice), SDMT, MMSE, and verbal fluency. The average time between the mPACC and the remote digital assessments was 182 days.

#### 2.5.4 Statistical analysis

Multiple regression analyses were carried out between PET measures, MTL subregional atrophy and unsupervised digital remote memory tests in R (version 4.1.2; www.r‐project.org; R Core Team, 2022). All models were adjusted for age, sex, and intracranial volume (for models including volumes). Results were corrected for multiple comparisons using false discovery rate correction (*p* < 0.05) where appropriate. Robust regression models were estimated using iteratively re‐weighted least squares (ILRS) using the MASS package (rlm function).

We extracted participant‐specific mPACC slopes from linear mixed‐effects models with random intercepts and slopes (using the lme4 package for R) and mPACC score as the outcome and time (visit number) as the predictor (on average 3.4 mPACC timepoints per participant across up to 5 years). These participant‐specific slopes were used as outcomes in a second set of linear regression models with plasma p‐tau217 and remote memory measures as predictors while adjusting for age, sex, and years of education. For comparison, we also fit basic models using only the covariates, without cognitive markers or biomarkers. Models were evaluated using the Akaike information criterion (AIC), where a difference of 2 or greater is considered significant.^41^ Retest reliability was assessed for 2 individual sessions (first and second session of the respective test; on average after 7 weeks) and for 2 averaged sessions (mean of first and second session of the respective test vs. mean of third and fourth session of the respective test; on average after 13 weeks). Intraclass correlation coefficients (ICCs) and their 95% confident intervals were calculated based on single rater, absolute‐agreement, 2‐way random‐effects model. We consider ICC values less than 0.5 indicative of poor reliability, values between 0.5 and 0.75 indicative for moderate reliability, values between 0.75 and 0.9 indicative for good reliability, and values greater than 0.90 indicative for excellent reliability.^42^

## 3 RESULTS

### 3.1 Participant sample

Here, we considered the 160 study participants who opted to participate in the add‐on smartphone‐based study. Of those 160, 38 did not enroll within the mobile app, another 19 enrolled but did not complete a full test session, and 3 were excluded due to no valid test sessions following quality assurance as described above. As a result, 100 individuals who contributed at least 1 valid test session of the MDT‐OS (see Table 1 and flow chart in Figure S1) and 66 participants who contributed at least 1 valid test session of the MDT‐OS and the ORR task (see Table S1) were included. Following initial filtering, we thus analyzed data from 49 CU Aβ−, 28 CU Aβ+, 9 MCI Aβ−, and 14 MCI Aβ+ in the MDT‐OS and from 31 CU Aβ−, 19 CU Aβ+, 7 MCI Aβ−, and 9 MCI Aβ+ in the ORR‐DR.

**TABLE 1 alz13919-tbl-0001:** Participant demographics of the entire sample.

Parameter	CU Aβ− (*N* = 49)	CU Aβ+ (*N* = 28)	MCI Aβ− (*N* = 9)	MCI Aβ+ (*N* = 14)	Total (*N* = 100)
Age (years)	60.6 (10.9)	69.8 (8.4)	64 (11.9)	67.8 (4.3)	64.5 (10.4)
Education (years)	13.5 (3.3)	12.9 (3.3)	12.7 (2.7)	12.5 (3.8)	13.1 (3.3)
Sex (% female)	53.1%	57.1%	55.6%	35.7%	52%
MMSE	29 (1.2)	28.6 (1.6)	27 (2.1)	27.7 (1.5)	28.5 (1.6)

*Note*: Displayed are mean values (standard deviations) unless otherwise stated.

Abbreviations: CU, cognitively unimpaired; MCI, mild cognitive impairment; MMSE, Mini‐Mental State Examination; *N*, number of participants.

### 3.2 Older participants show high acceptability of smartphone‐based assessments

First, we were interested in the acceptability of the remote smartphone‐based assessments. A total of 120 participants completed a questionnaire about their phone usage. Out of those, 88% owned a smartphone, 64% reported they would download apps by themselves, and 21% with assistance. Participants reported that they had been using a smartphone for 8.2 years on average (SD: 4.5 years, range: 1–25 years), and spend 1.7 h per day using it (SD: 1.6 h, range: 1–10 h).

As reported above, 160 individuals were recruited into the add‐on smartphone study. The most frequent reason not to participate in the smartphone‐based study was lack of time (too many assessments; 13%), followed by unfamiliarity with smartphone use and insecurity about their capabilities (7%), as well as not owning a smartphone with Internet connection (7%). Seventy‐three percent did not further specify any reasons.

Across both cognitive tests, participants reported high average concentration levels (MDT‐OS = 3.9; ORR = 3.9; scale 1–5, which translates to good concentration) during the task as well as medium subjectively rated task difficulty (MDT = 3.1; ORR = 3.4; scale 1–5, which translates to moderate subjectively rated performance). Both estimates were very similar across diagnostic groups (group average range in concentration from 3.7 to 4.1 and in subjective task difficulty from 2.9 to 3.5). However, as expected, the second phase of the task (i.e., 2‐back or delayed retrieval compared to 1‐back or immediate retrieval) was perceived as considerably more difficult (phase 1: 3.7; phase 2: 2.7). Across both tasks, 88% of participants reported no distractions during the task and distraction rates were higher in cognitively unimpaired individuals (on average 14% across both tasks) compared to MCI patients (on average 6% across both tasks).

Following filtering, the time between encoding and retrieval in the ORR test was on average 96 minutes (SD = 44). Mobile devices had a screen diagonal between 10.2 and 24.6 cm (mean 13.2 cm, SD = 3.4). While 91% of participants completed the test sessions on a smartphone, 9% used a tablet. Regarding operating systems, 59% used iOS devices, 35% used Android devices, and for 6% the operating system was unknown.

**FIGURE 3:**
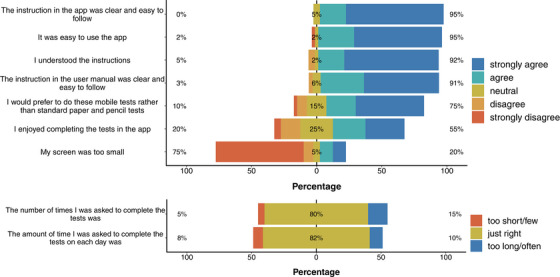
Acceptability and user experience. Results from telephone‐based interviews with 38 participants focusing on their overall experience with the remote and unsupervised study.

We approached 38 participants for a telephone‐based interview to learn about their experience with the study (see Figure 3). Over 90% of them found the tasks and instructions easy to understand, and the app easy to use. Seventy‐six percent said they would prefer the mobile tests over in‐person paper‐and‐pencil tests. Seventy‐nine percent did not experience their device's screen as too small to see all the details in the tasks. Eighty‐four percent found the number of tests and their duration just right, and 86% rated their experience using the app 7 or higher on a 10‐point scale. Out of the 38 participants who completed the phone interview, 36 had finished the study and only 2 participants had dropped out of the study before the end. While those 2 similarly thought that the app was easy to use and the instructions were clear, 1 thought that the screen was too small and both did not enjoy completing the tests in the app (disagree and neutral). One of them found the amount and number of times was just right, while the other rated them as too long and too often (see Figure S2).

Finally, we compared individuals that were not interested in participating, those that agreed to participate but never enrolled in the mobile app, those that enrolled but never completed a valid test session and those that contributed valid test sessions regarding key characteristics to further understand the participating sample. We found that those participants who provided valid test sessions were on average younger than those from all other groups (*F* = 8.6, *p* < 0.001; M_NotInterested_ = 72.5 years (SD 6.38), M_NotEnrolled _= 72 years (SD = 7.36), M_NoTests _= 69.2 years (SD = 8.4), M_ValidTests _= 64.5 (SD = 10.4)). Further, 67% of those that were not interested in participating were MCI patients, while only 26% of individuals across all other groups had an MCI diagnosis. There was no difference in CSF Aβ42/40 levels across groups.

We found no differences between the groups regarding their daily use of smartphones (*F* = 1.43, *p* = 0.239) and the years they have been using smartphones (*F* = 0.44, *p* = 0.724). However, 62.5% of those that were not interested in participating or never enrolled in the app had either never installed an app before or had done so only with help. In contrast, 69% of those who enrolled in the app and/or provided valid data had already installed apps on their own.

### 3.3 Unsupervised memory assessments via mobile devices reflect in‐clinic cognitive scores

Next, we were interested whether the performance in the unsupervised and remote memory assessments was associated with traditional in‐clinic cognitive measures. To understand the relationship with demographics and potential confounds, we first calculated multiple regression models with age, sex, years of education, device type (smartphone or tablet), and operating system to identify the associations with both memory paradigms. In addition, we included time‐to‐retrieval, that is, the time between memory encoding and retrieval, as a covariate for the ORR‐DR. For MDT‐OS, age and sex were significant predictors for the object, and age and device type were a significant predictor for the scene condition where higher age, female sex, and tablet usage were associated with worse task performance (MDT‐O: *β*_age_ = −0.008, *p *< 0.001; *β*_sex_ = −0.08, *p* = 0.045; *β*_education_ = 0.006, *p* = 0.287; *β*_device type_ = −0.13, *p* = 0.0524; *β*_OS_ = −0.05, *p* = 0.209; MDT‐S: *β*_age_ = −0.009, *p *< 0.001; *β*_sex_ = −0.08, *p *= 0.062; *β*_education_ = 0.008, *p *= 0.230; *β*_device type_ = −0.18, *p* = 0.015; *β*_OS_ = −0.05, *p* = 0.240). For the ORR‐DR, only age was associated with task performance (*β*_age_ = −0.22, *p *< 0.001; *β*_sex_ = −0.24, *p *= 0.814; *β*_education_ = 0.08, *p *= 0.617; *β*_delay_ = −0.63, *p *= 0.358; *β*_device type_ = −1.59, *p* = 0.427; *β*_OS_ = −1.88, *p* = 0.065).

**FIGURE 4:**
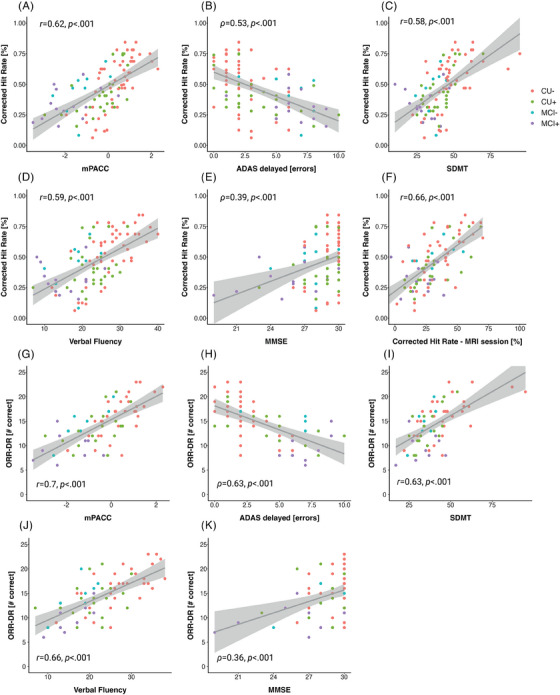
Construct validity of the MDT‐OS and the ORR. Scatter plots showing relationships of the MDT‐OS Corrected hit rate with the (A) modified Preclinical Alzheimer's Cognitive Composite, (B) errors in the delayed 10‐word list recall test from ADAS‐cog, (C) Symbol Digit Modalities Test, (D) animal fluency, (E) Mini‐Mental State Examination as well as the (F) Mnemonic Discrimination Task for Objects and Scenes Corrected Hit Rate from an on‐site based task version that was performed during an MRI scan. Furthermore, scatter plots show relationships of the ORR‐DR with the (G) modified Preclinical Alzheimer's Cognitive Composite, (H) errors in the delayed 10‐word list recall test from ADAS‐cog, (I) Symbol Digit Modalities Test, (J) animal fluency, and the (K) Mini‐Mental State Examination. ADAS, Alzheimer's Disease Assessment Scale; ADAS‐cog, Alzheimer's Disease Assessment Scale—Cognitive Subscale; MDT‐OS; mnemonic discrimination test for objects and scenes; ORR, Objects‐In‐Room Recall; ORR‐DR, ORR delayed recall score.

Next, we assessed the construct validity of the outcomes of the unsupervised memory assessments by analyzing the relationship with several in‐clinic and supervised cognitive assessments (see Figure 4). This included an in‐scanner version of the MDT‐OS which was completed by the same participants during an Functional Magnetic Resonance Imaging scan. While this was a similar task (MDT‐OS), task administration (controller button presses instead of a touch screen) and environment (lying in an MRI scanner vs. completing tasks at home) differed compared to the remote unsupervised setting using a smartphone. Pearson correlation coefficients revealed a strong relationship between the MDT‐OS corrected hit rate across both settings (*r *= 0.66, *p *< 0.001; MDT‐O: *r *= 0.52, *p *< 0.001; MDT‐S: *r *= 0.48, *p *< 0.001). Next, we were interested whether MDT‐OS and ORR‐DR were associated with an in‐clinic measure of memory (ADAS delayed recall) and a composite score that has been shown to be sensitive to early AD (mPACC) as well as the SDMT, verbal fluency and the MMSE. Due to ceiling effects of ADAS delayed recall and the MMSE in early AD, we assessed Spearman rank correlation rho. All measures were significantly associated with the MDT‐OS corrected hit rates (*ADASdelayed*: *ρ* = 0.53, *p *< 0.001; MDT‐O: *ρ* = 0.54, *p *< 0.001; MDT‐S: *ρ* = 0.44, *p *< 0.001; *MMSE*: *ρ* = 0.39, *p *< 0.001; MDT‐O: *ρ* = 0.37, *p *< 0.001; MDT‐S: *r *= 0.33, *p *< 0.001; *VerbalFluency*: *r *= 0.59, *p *< 0.001; MDT‐O: *r *= 0.55, *p *< 0.001; MDT‐S: *r *= 0.52, *p *< 0.001; *SDMT*: *r *= 0.58, *p *< 0.001; MDT‐O: *r *= 0.54, *p *< 0.001; MDT‐S: *r *= 0.50, *p *< 0.001). Similarly, ORR‐DR performance was strongly associated with ADAS delayed recall and also associated with all other outcomes (*ADASdelayed*: *ρ* = 0.63, *p *< 0.001; *MMSE*: *ρ* = 0.36, *p *< 0.001; *VerbalFluency*: *r *= 0.66, *p *< 0.001; *SDMT*: *r *= 0.63, *p *< 0.001).

The mPACC score was associated with MDT‐OS (mPACC: *r *= 0.62, *p *< 0.001; MDT‐O: *r *= 0.58, *p *< 0.001; MDT‐S: *r *= 0.54, *p *< 0.001) and ORR delayed retrieval performance (*r *= 0.7, *p *< 0.001). All relationships above survived corrections for multiple comparisons using False Discovery Rate (FDR) correction.

### 3.4 Digital memory scores are associated with measures of AD pathology and MTL atrophy

**FIGURE 5:**
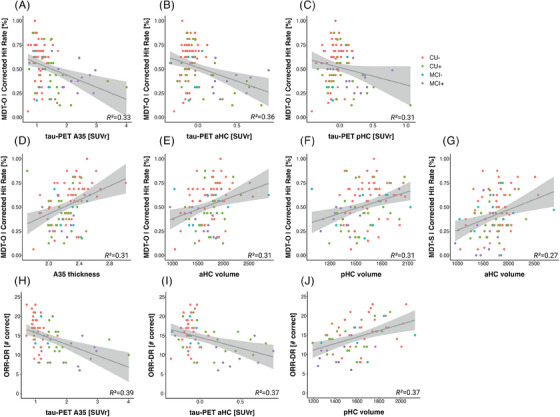
Criterion validity of the MDT‐OS and the ORR‐DR. Scatter plots showing relationships between the MDT‐O and tau‐PET uptake in (A) area 35, (B) the anterior and (C) posterior hippocampus, as well as cortical thickness in area 35 (D) and anterior (E) and posterior hippocampal volume (F). Panel G shows the relationship of the MDT‐S with anterior hippocampal volume. Finally, panel (H), (I), and (J) illustrate relationships of the ORR‐DR and tau‐PET uptake in area 35 and the anterior hippocampus as well as the volume of the posterior hippocampus. A35, area 35; aHC, anterior hippocampus; pHC, posterior hippocampus; MDT‐OS; Mnemonic Discrimination Task for Objects and Scenes; ORR‐DR, Object‐In‐Room Recall–delayed recall; PET, positron emission tomography.

Next, we were interested whether memory scores from remote and unsupervised assessments were associated with measures of AD pathology, namely, Aβ and tau pathology, as well as measures of atrophy (see Figure 5). Both memory tasks have been designed to tax MTL‐dependent memory processes.^3^, ^11^, ^13^ Thus, we were interested in the relationship with measures of tau pathology representing early disease stages. Following recent findings on the relationship of memory performance with measures of MTL tau pathology and atrophy,^4^, ^43^, ^44^ we selected tau‐PET SUVr in area 35 and the anterior and posterior hippocampus as well as median thickness of area 35 and the volume of the anterior and posterior hippocampus. Regarding Aβ, we selected SUVr in the precuneus representing early Aβ deposition.^45^

Regarding tau‐PET, MDT‐O, but not MDT‐S, was significantly associated with area 35 tau‐PET SUVr (MDT‐O: *β* = −0.09, SE = 0.03, *p *= 0.007; MDT‐S: *β* = −0.04, SE = 0.04, *p *= 0.229), anterior hippocampal tau‐PET SUVr (MDT‐O: *β* = −0.24, SE = 0.07, *p *< 0.001; MDT‐S: *β* = −0.019, SE = 0.08, *p *= 0.813), and posterior hippocampal tau‐PET SUVr when accounting for participant age and sex (MDT‐O: *β* = −0.17, SE = 0.08, *p *= 0.03; MDT‐S: *β* = −0.001, SE = 0.09, *p *= 0.989). Likewise, ORR‐DR performance was significantly associated with area 35 tau‐PET SUVr (*β* = −2.3, SE = 0.7, *p *= 0.002) and anterior (*β* = −4.44, SE = 1.53, *p *= 0.005) but not posterior hippocampal tau‐PET SUVr (*β* = −2.99, SE = 1.86, *p *= 0.113) when accounting for participant age, sex and time between encoding and retrieval. These relationships survived corrections for multiple comparisons using FDR correction. However, when additionally accounting for Aβ‐PET SUVr in precuneus, only the relationship between the MDT‐O and the anterior hippocampus remained statistically significant (MDT‐O: *β* = −0.22, SE = 0.09, *p *= 0.013) while the relationship of the MDT‐O with area 35 and posterior hippocampal tau‐PET weakened (area 35: *β* = −0.07, SE = 0.04, *p *= 0.113; posterior hippocampus: *β* = −0.11, SE = 0.1, *p *= 0.242) as did the relationship of the ORR‐DR and area 35 and anterior hippocampal tau‐PET SUVr (area 35: *β* = −1.54, SE = 0.97, *p *= 0.117; anterior hippocampus: *β* = −2.37, SE = 2.02, *p *= 0.245)

Similarly, regarding Aβ‐PET, MDT‐O, and ORR‐DR performance, but not MDT‐S, were significantly associated with Aβ‐PET SUVr in precuneus (MDT‐O: *β* = −0.18, SE = 0.08, *p *= 0.026; MDT‐S: *β* = −0.08, SE = 0.09, *p *= 0.343; ORR: *β* = −5.66, SE = 01.85, *p *= 0.003). However, the Aβ‐PET SUVr relationships did not hold when accounting for area 35 tau‐PET SUVR (all *p*‐values > 0.26).

With respect to MTL measures of atrophy, MDT‐O was significantly associated with area 35 thickness as well as anterior and posterior hippocampal volume (area 35: *β* = 0.22, SE = 0.11, *p *= 0.044 [robust regression: *F* = 5.34, *p *= 0.022]; anterior hippocampus: *β* = 301.3, SE = 150.8, *p *= 0.049; posterior hippocampus: *β* = 218, SE = 108, *p *= 0.046), while MDT‐S was only associated with anterior hippocampal volume (anterior hippocampus: *β* = 420.4, SE = 134.4, *p *= 0.002, A35: *β* = 0.17, SE = 0.1, *p *= 0.081, posterior hippocampus: *β* = 190.7, SE = 99.2, *p *= 0.058) when accounting for age, sex, and intracranial volume. ORR‐DR performance was significantly associated with posterior hippocampal volume (*β* = 15, SE = 6, *p *= 0.015) but neither anterior hippocampal volume (*β* = 15.6, SE = 8.03, *p *= 0.057) nor area 35 thickness (*β* = 0.001, SE = 0.006, *p *= 0.076) when accounting for age, sex, intracranial volume and time between encoding and retrieval. The relationships of the MDT‐O and MTL atrophy measures did not survive corrections for multiple comparisons using FDR correction. Taken together, both memory tasks showed sensitivity to measures of AD pathology as well as MTL atrophy as a measure of neurodegeneration.

### 3.5 Combination of blood‐based biomarker and digital cognitive marker predicts future cognitive decline

Next, we examined whether baseline scores from unsupervised memory assessments were associated with future cognitive decline rates in the mPACC and whether a combination of a blood‐based biomarker and digital cognitive markers would outperform individual measures in predicting cognitive decline. Recent work has shown that plasma p‐tau217 was associated with cognitive decline as well as disease progression.^46^, ^47^ To that end, we derived participant‐specific mPACC slopes from linear mixed‐effects model with random intercepts and slopes and ran hierarchical linear regression models with plasma p‐tau217 and remote memory measures as predictors to test which model best predicted mPACC decline across 5 years.

Adjusting for covariates, cognitive outcomes for MDT‐S and ORR‐DR as well as p‐tau217 but not MDT‐O were significantly associated with mPACC slopes (see Tables 2 and 3, see also Table S2 for individual longitudinal linear mixed‐effects models for each marker).

**TABLE 2:** Results of multiple regression models in the entire sample (*n* = 86^a^) predicting future cognitive decline including baseline plasma p‐tau217 and digital remote markers as predictors, demographic covariates, and mPACC slopes as an outcome.

	Null model			Digital cognitive marker			BBM			Digital cognitive marker + BBM		
Predictor	Estimate	CI	*p*‐Value	Estimate	CI	*p*‐Value	Estimate	CI	*p*‐Value	Estimate	CI	*p*‐Value
(intercept)	0.317	0.203–0.432	**<0.001**	0.149	0.015–0.284	**0.029**	0.316	0.205 to 0.426	**<0.001**	0.179	0.062 to 0.296	**0.003**
Age (years)	−0.005	−0.006 to −0.004	**<0.001**	−0.003	−0.005 to −0.002	**<0.001**	−0.004	−0.005 to −0.003	**<0.001**	−0.003	−0.004 to −0.001	**<0.001**
Sex (female)	0.031	0.004 to 0.059	**0.023**	0.050	0.023 to 0.077	**<0.001**	0.029	0.002 to 0.055	**0.032**	0.044	0.019 to 0.070	**0.001**
Education (years)	0.000	−0.004 to 0.004	0.975	−0.003	−0.007 to 0.001	0.192	−0.001	−0.005 to 0.003	0.649	−0.004	−0.008 to 0.000	0.069
MDT‐O				0.048	−0.034 to 0.130	0.251						
MDT‐S				0.149	0.073 to 0.225	**<0.001**				0.174	0.111 to 0.237	**<0.001**
p‐tau217							−0.024	−0.034 to −0.014	**<0.001**	−0.024	−0.034 to −0.014	**<0.001**
Observations	292			292			292			292		
*R* ^2^/*R* ^2^ adj	0.180/0.172			0.252/0.239			0.235/0.225			0.306/0.294		
AIC	−429.038			−451.968			−447.381			−473.811		

Abbreviations: AIC, Akaike information criterion; BBM, blood‐based biomarker; CI, confidence interval; MDT‐O, Mnemonic Discrimination Task for Objects; MDT‐S, Mnemonic Discrimination Task for Scenes; mPACC, modified Preclinical Alzheimer's Cognitive Composite.

Bold values significance *p* < 0.05.

^a^ *Note* that 14 participants did not have p‐tau217 levels available.

**TABLE 3:** Results of multiple regression models in a restricted sample (*n* = 55^a^) where both digital markers were completed predicting future cognitive decline including baseline plasma p‐tau217 and digital remote cognitive markers as predictors, demographic covariates, and mPACC slopes as an outcome.

	Null model			Digital cognitive marker			BBM			Digital cognitive marker + BBM		
Predictor	Estimate	CI	*p*‐Value	Estimate	CI	*p*‐Value	Estimate	CI	*p*‐Value	Estimate	CI	*p*‐Value
(intercept)	0.328	0.175 to 0.482	**<0.001**	−0.133	−0.315 to 0.049	0.151	0.314	0.166 to 0.461	**<0.001**	−0.106	−0.275 to 0.064	0.220
Age (years)	−0.005	−0.007 to −0.003	**<0.001**	−0.001	−0.003 to 0.001	0.475	−0.004	−0.005 to −0.002	**<0.001**	0.000	−0.002 to 0.002	0.858
Sex (female)	0.043	0.005 to 0.080	**0.027**	0.059	0.025 to 0.094	**0.001**	0.033	−0.003 to 0.070	0.076	0.048	0.015 to 0.080	**0.004**
Education (years)	−0.002	−0.007 to 0.004	0.582	−0.008	−0.013 to −0.003	**0.004**	−0.003	−0.008 to 0.003	0.338	−0.009	−0.014 to −0.004	**<0.001**
MDT‐O				0.089	−0.021 to 0.198	0.113						
MDT‐S				0.102	0.009 to 0.195	**0.033**				0.167	0.086 to 0.249	**<0.001**
ORR‐DR				0.013	0.007 to 0.018	**<0.001**				0.012	0.007 to 0.017	**<0.001**
p‐tau217							−0.028	−0.041 to −0.014	**<0.001**	−0.028	−0.041 to −0.016	**<0.001**
Observations	187			187			187			187		
*R* ^2^/*R* ^2^ adj	0.151/0.138			0.358/0.337			0.219/0.201			0.417/0.398		
AIC	−233.573			−279.850			−246.965			−297.803		

Abbreviations: AIC, Akaike information criterion; BBM, blood‐based biomarker; CI, confidence interval; MDT‐O, Mnemonic Discrimination Task for Objects; MDT‐S, Mnemonic Discrimination Task for Scenes; mPACC, modified Preclinical Alzheimer's Cognitive Composite; ORR‐DR, Object‐In‐Room Recall–Delayed Recall.

Bold values significance *p* < 0.05.

^a^ *Note* that 11 participants did not have p‐tau217 levels available.

We next aimed to define an optimal combination to predict mPACC slopes. The best combination model for prediction of mPACC slopes (i.e., the model with the lowest AIC) included plasma p‐tau217 (*β* [SE]  =  −0.024 [0.005]; *p*  <  0.001), MDT‐S (*β* [SE]  =  0.174 [0.032]; *p*  <  0.001), age (with higher age associated with worse mPACC slopes; *β* [SE]  =  −0.003 [0.001]; *p*  =   <  0.001), sex (with male sex associated with worse mPACC slopes; *β* [SE]  =  0.044 [0.013]; *p*  <  0.001), and education (*β* [SE]  =  −0.004 [0.002]; *p *= 0.069; AIC for the overall model: −474; *R* ^2^  =  0.31, see Table 2 “Digital Cognitive Marker + BBM”). This model was also better than both the Digital Cognitive Marker and the BBM model as indicated by a lower AIC.

Similarly, in a smaller sample of individuals that completed both the MDT‐OS and the ORR‐DR (see Table S1 for sample characteristics), the best combination model for prediction of mPACC slopes included plasma p‐tau217 (*β* [SE]  =  −0.03 [0.006]; *p*  <  0.001), ORR‐DR (*β* [SE]  =  0.012 [0.003]; *p*  <  0.001), MDT‐S (*β* [SE]  =  0.167 [0.041]; *p*  <  0.001), age (*β* [SE]  =  0.000 [0.001]; *p*  =  0.858), sex (with male sex associated with worse mPACC slopes; *β* [SE]  =  0.048 [0.016]; *p*  =  0.004), and education (with higher education associated with worse mPACC slopes; (*β* [SE]  =  −0.009 [0.003]; *p *  < 0.001; AIC for the overall model: −298; *R* ^2^  =  0.42, see Table 3 “Digital Cognitive Marker + BBM”). This model was also better than both the Digital Cognitive Marker and the BBM model with respect to lower AIC.

### 3.6 Remote and unsupervised short assessments show moderate‐to‐good retest reliability

Finally, we were interested in retest reliability of remote and unsupervised assessments given that unsupervised and remote memory assessments are well suited to assess longitudinal memory trajectories. To that end, we limited the dataset to individuals who had at least 4 sessions completed (*n* = 73 and *n* = 37 completed repeated assessments across 4 sessions of the MDT‐OS and ORR respectively) and calculated 2‐way random ICC for 2 scenarios. Regarding a scenario where single test sessions would be used, we calculated the ICC between the first and second session of a respective test and regarding a scenario where the average between 2 sessions would be used, we calculated the ICC between the average of the first and second session of a respective test and the average the third and fourth session of a respective test. A single session of MDT‐OS (ICC of 0.65, 95% CI [0.5, 0.77]; MDT‐O: 0.58 [CI 0.4, 0.71]; MDT‐S: 0.52 [CI 0.33, 0.67]; *n* = 73) and a single session of ORR (ICC of 0.67 [CI 0.41, 0.8]; *n *= 37) both showed moderate retest reliability. In the second scenario, the average of 2 test sessions of MDT‐OS (ICC of 0.83 [CI 0.7, 0.89]; MDT‐O: 0.65 [CI 0.49, 0.76]; MDT‐S: 0.68 [CI 0.53, 0.78]), as well as the average of two test sessions of ORR (ICC of 0.79 [CI 0.63, 0.89]) showed good retest reliability.

## 4 DISCUSSION

We found good construct validity of remote and unsupervised digital memory assessments when comparing them with in‐clinic traditional neuropsychological assessments, and that baseline performance in the remote memory assessments was associated with decline in the mPACC score. The model that best predicted cognitive decline in the mPACC included a combination of plasma p‐tau217 and MDT‐S. In a sub‐sample of individuals where both digital cognitive markers were completed, the model that best predicted future cognitive decline in the mPACC also included the ORR‐DR. Retest reliability was moderate‐to‐good when repeating the tests utilizing parallel test versions. In addition, digital memory assessments were significantly associated with tau‐PET as well as downstream MRI measures of MTL atrophy. Finally, the onboarding into the study as well as the unsupervised completion of memory tasks itself was rated positively by participants.

### 4.1 Older adults and patients were able to complete unsupervised digital assessments

There exist stereotypes regarding older adults’ and patients’ familiarity with smartphones and tablet computers and their willingness to participate in unsupervised studies using digital devices. However, recent work has shown that, while older adults were indeed less familiar with technology, many older participants decided to participate in remote studies and showed exceptional adherence when studies were planned thoughtful and included user‐centered design.^48^ In line with this, we found that a large majority of older participants owned a mobile device and downloaded apps by themselves or with help. However, we found that MCI and older age were more common in individuals who were not interested in participating or did not contribute complete datasets. Many of those who did not participate indicated that they had either never installed an app before or only with help, while the majority of participants who contributed complete datasets had already installed apps on their own. While only 10% were not interested in participating in the remote assessments, more than 20% of individuals who initially agreed to participate did never enroll within the app. Therefore, there seems to be a large potential to encourage participation by offering support to those with MCI, that are older and those with less experience with smartphones as has been demonstrated in earlier work.^48^

Participants rated both remote memory paradigms as challenging but not too difficult. A large majority found the instructions clear and the application easy to use, and the app was overall rated at least 7 out of 10 by more than 85% of participants. This indicates that the app was easy to work with for most participants. Importantly, however, these interviews almost exclusively included participants who had finished the study after 1 year which might have led to biased results. Future studies thus need to incorporate usability interviews earlier in the study schedule.

### 4.2 Remote and unsupervised assessments reflect traditional neuropsychological assessments

When introducing a novel cognitive measure, construct validity needs to be assessed by comparing it against established neuropsychological measures of constructs it is intended to measure. This is particularly true for unsupervised and remote digital assessments that are completed in unstandardized environments of the participant's choice. Recent work using smartphone‐based assessments in samples of older participants could demonstrate high construct validity across various cognitive domains.^20^, ^21^, ^22^, ^49^, ^50^ Both the ORR and the MDT‐OS measure visual memory, which makes it difficult to cheat, as would be possible in verbal memory tasks, where participants could, for example, take notes. While the ORR specifically aims to measure delayed memory, the MDT‐OS measures precision memory in an n‐back task design known to also rely on attentional and executive processes.^51^ We found that the ORR‐DR and MDT‐OS were strongly associated with ADAS delayed recall, SDMT, and verbal fluency and less so with the MMSE. The strongest relationship for both measures was found with the mPACC. These findings support earlier work using the neotiv memory tasks in samples with CU and MCI patients.^21^, ^28^ Furthermore, baseline performance in the MDT‐S and the ORR‐DR was associated with future decline in the mPACC in this study, demonstrating first evidence of its prognostic validity regarding cognitive decline.

While high construct validity indicates that the tasks were successfully designed to measure memory function, it also indicates that study participants were successful in completing the tests in an appropriate environment. While we do not know details about the individual test environment, our findings indicate that it was generally an undisturbed environment, as 88% of all sessions were reported without any distractions. Interestingly, our findings show that CU individuals reported more distractions compared to MCI patients, in line with earlier research.^52^

### 4.3 Remote and unsupervised assessments are sensitive to measures of AD pathology

Both the MDT‐OS and the ORR are considered MTL‐dependent tasks^3^, ^11^, ^13^, ^15^, ^16^ and earlier work has already linked performance in the MDT‐OS with fluid and imaging measures of AD pathology.^3^, ^13^ While the MDT‐O is associated with an object pathway including the perirhinal cortex (including area 35) and the anterior‐lateral entorhinal cortex, the MDT‐S targets a scene pathway including the parahippocampal cortex and the posterior‐medial entorhinal cortex.^3^, ^11^, ^13^ Elevated tau‐PET signal in early disease stages can be found in perirhinal cortex and the anterior‐lateral entorhinal cortex before it progresses toward the hippocampus.^4^ Thus, we would expect relationships and effects of tau pathology specifically on object memory as we have seen in earlier work.^3^, ^13^ In line with these earlier findings, we found that MDT‐O and ORR‐DR were both significantly associated with tau‐PET measures from the hippocampus and area 35, and there was no such relationship for the MDT‐S.^13^ However, these effects weakened, and several became non‐significant when we added a measure of amyloid burden to the models. While this is in part expected given the high correlation between tau and amyloid burden, future analyses with bigger sample sizes need to confirm these relationships. Finally, we found that remote digital assessments were associated with MTL atrophy, which is considered a downstream effect of AD pathology. Area 35 and the hippocampus are among the earliest sites of atrophy in AD.^4^, ^53^, ^54^ While the MDT‐OS was associated with atrophy of the hippocampus and area 35, ORR‐DR was only associated with posterior hippocampal atrophy. Taken together, this shows that both tasks depend on the functional integrity of the MTL, and that task performance is affected by early accumulation of AD pathology.

### 4.4 Potential of remote assessments for case finding, monitoring, and prognosis of cognitive impairment in AD

Remote unsupervised digital cognitive assessments hold promise for case finding in healthcare and clinical trials, but also for longitudinal cognitive monitoring and even prognosis of cognitive decline and disease progression. Recent work showed that outcomes from remote assessments can help to identify MCI patients^28^ and potentially even Aβ‐positive CU participants.^19^, ^26^ Regarding prognosis, recent work has shown that plasma biomarkers for tau pathology are associated with future cognitive decline,^46^ and in combination with brief in‐clinic pen‐and‐paper cognitive tests, can identify MCI patients who are likely to progress toward dementia.^47^ Similarly, Tsoy and colleagues recently showed that a combination of plasma biomarkers and brief in‐clinic digital cognitive assessments could predict Aβ‐positivity and were associated with concurrent disease severity as well as future functional decline.^55^ High construct validity and first evidence of prognostic validity regarding cognitive decline in the mPACC suggest that remote memory assessments may also have potential utility in prognosis. Indeed, our analysis comparing models with plasma p‐tau217 against a model combining plasma p‐tau217 and remote digital memory assessments suggests that incorporating remote memory assessments can significantly contribute to the prediction of future decline. Moreover, the moderate‐to‐good retest reliability of both memory assessments, coupled with the minimal practice effects due to the utilization of parallel test sets^24^ hints that these tests might prove beneficial in monitoring cognitive change over time. While retest reliability was moderate when using one single assessment, it was good when averaging across only two sessions. Future studies need to investigate whether longitudinal trajectories derived from remote and unsupervised cognitive assessments can capture subtle cognitive decline.

We need to carefully consider some limitations to this study. First, the modest sample size, particularly for participants who completed both memory paradigms. Second, our implementation of the ORR did not strictly enforce adherence to the planned retrieval delay intervals, which led some individuals to perform recall assessments after prolonged delays. Given the impact of delay length on task performance, we excluded sessions with significantly extended delay periods (more than 240 minutes) resulting in substantial reduction of test sessions in this study. Thus, future implementations of this task, and remote and unsupervised assessments of long‐term memory in general, should make it easier for participants and patients to integrate remote and repeated tests into their everyday life, while still enforcing minimized delay periods.

## 5 CONCLUSION

Our results demonstrate that unsupervised and remote digital memory assessments could effectively become a valuable tool in the diagnosis and prognosis of AD, conceivably in combination with plasma biomarkers.

## CONFLICT OF INTEREST STATEMENT

O.H. has acquired research support (for the institution) from ADx, AVID Radiopharmaceuticals, Biogen, Eli Lilly, Eisai, Fujirebio, GE Healthcare, Pfizer, and Roche. In the past 2 years, he has received consultancy/speaker fees from AC Immune, Amylyx, Alzpath, BioArctic, Biogen, Cerveau, Eisai, Eli Lilly, Fujirebio, Merck, Novartis, Novo Nordisk, Roche, Sanofi and Siemens. S.P. has acquired research support (for the institution) from ki elements/ADDF and Avid. In the past 2 years, he has received consultancy/speaker fees from Bioarctic, Biogen, Eisai, Lilly, and Roche. E.D. reports personal fees from Biogen, Roche, Lilly, Eisai and UCL Consultancy as well as non‐financial support from Rox Health. D.B. and E.D. are scientific co‐founders of neotiv GmbH and own company shares. The other authors report no competing interests. Author disclosures are available in the supporting information.

## CONSENT STATEMENT

All human participants provided informed consent to participate.

## Supporting information

ICMJE Disclosure Form

Supporting Information

## Data Availability

Anonymized data will be shared by request from a qualified academic investigator for the sole purpose of replicating procedures and results presented in the article and if data transfer is in agreement with EU legislation on the general data protection regulation and decisions by the Ethical Review Board of Sweden and Region Skåne, which should be regulated in a material transfer agreement.
