# Supplementary material for: Remote and unsupervised digital memory assessments can reliably detect cognitive impairment in Alzheimer's disease
Source: Alzheimers Dement. 2024 Jun 12;20(7):4775–91. doi: 10.1002/alz.13919 (PMC11247711; doi:10.1002/alz.13919)
Supplement: Supplementary file 2 — Supporting Information [file ALZ-20-4775-s001.pdf]

Remote and unsupervised digital memory assessments can reliably detect cognitive impairment in Alzheimer's disease

David Berron[1,2], Emil Olsson[1], Felix Andersson[3], Shorena Janelidze[1], Pontus Tideman[1,3], Emrah Düzel[2,3,4], Sebastian Palmqvist[1,5], Erik Stomrud[1,5], Oskar Hansson[1,5]

[1] *Clinical Memory Research Unit, Department of Clinical Sciences Malmö, Lund University, 223 62 Lund, Sweden*
[2] *German Center for Neurodegenerative Diseases, 39120 Magdeburg, Germany*
[3] *Institute for Cognitive Neurology and Dementia Research, Otto-von-Guericke University, Magdeburg, Germany*
[4] *Institute of Cognitive Neuroscience, University College London, United Kingdom*
[5] *Memory Clinic, Skåne University Hospital, 205 02 Malmö, Sweden*

Supplementary material

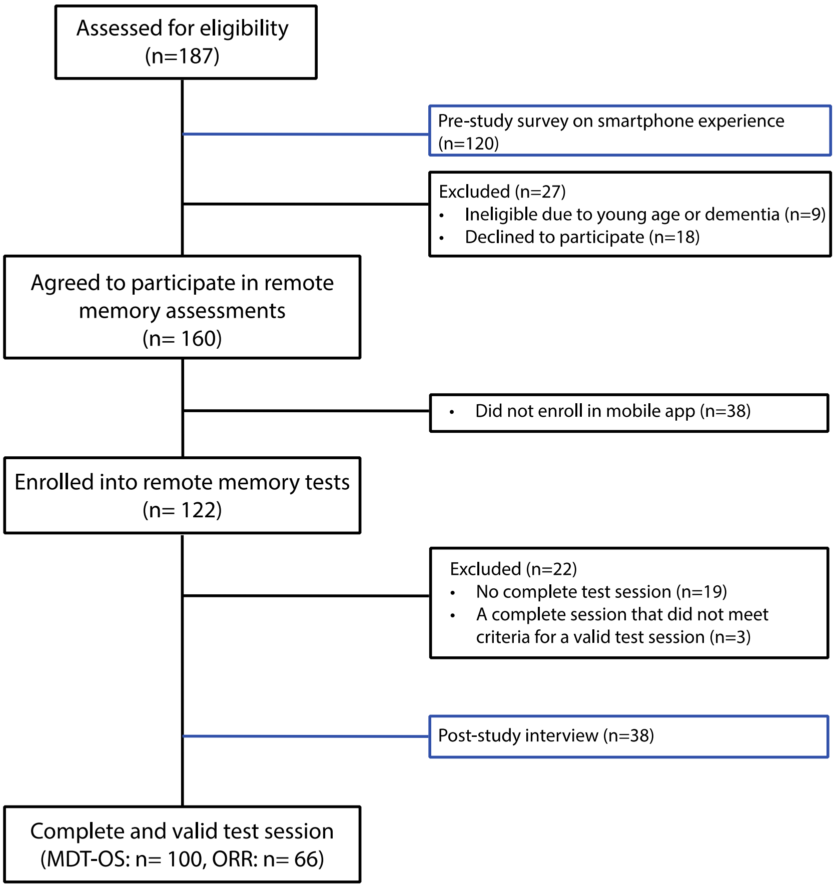

Supplementary Figure 1: Flow Chart

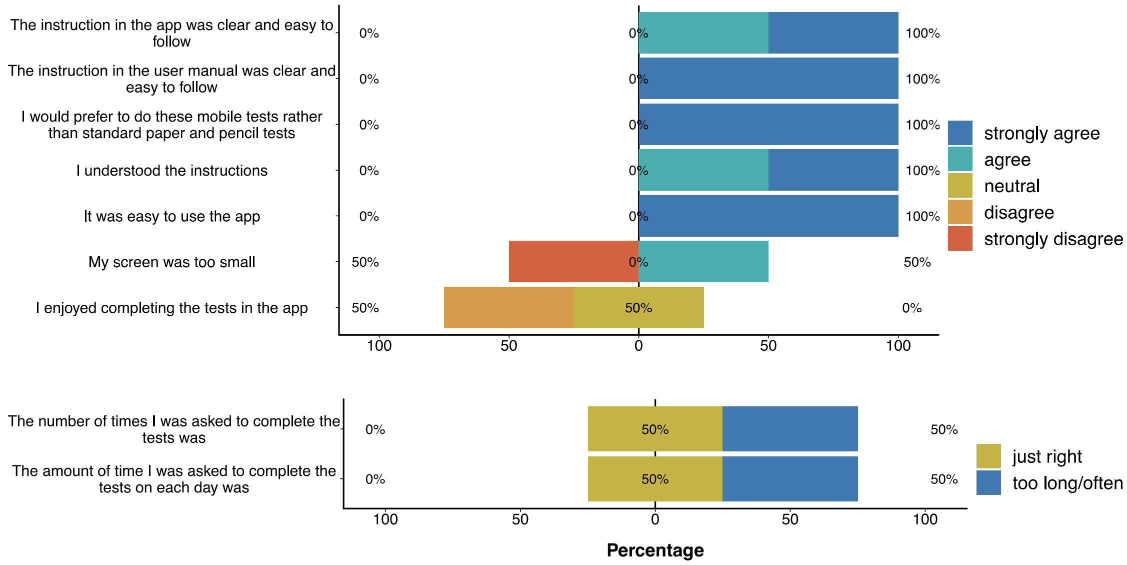

Supplementary Figure 2: Acceptability and User experience. Results from telephone-based interviews with two participants who did drop-out early focusing on their overall experience with the remote and unsupervised study.

Supplementary Table 1: Demographics of the sample that performed the MDT-OS and ORR-DR

	CU Aβ- (N=31)	CU Aβ+ (N=19)	MCI Aβ- (N=7)	MCI Aβ+ (N=9)	Total (N=66)
Age (years)	61.3 (10.9)	71.4 (7.4)	64 (13.1)	67.8 (4.3)	65.4 (10.4)
Education (years)	13.1 (3.1)	12.8 (3.6)	12.7 (3)	13.6 (4)	13 (3.3)
Sex (% female)	51.6 %	68.4 %	71.4 %	22.2 %	54.5 %
MMSE	29 (1.2)	28.3 (1.8)	26.4 (2.1)	27.6 (1.5)	28.3 (1.7)

Supplementary Table 2: Longitudinal random slope linear mixed models in the entire sample predicting future cognitive decline including baseline plasma and digital remote markers and years since baseline as predictors, demographic covariates, and mPACC slopes as an outcome. The term of interest is the interaction between baseline markers and years since baseline.

	**MDT-S**			**MDT-O**			**ORR-DR**			**p-tau217**		
Predictor	***Estimate***	***CI***	***p***	***Estimate***	***CI***	***p***	***Estimate***	***CI***	***p***	***Estimate***	***CI***	***p***
(Intercept)	0.519	-0.841 – 1.878	0.454	-0.383	-1.728 – 0.961	0.575	-1.218	-3.123 – 0.686	0.209	1.750	0.376 – 3.124	**0.013**
YearsSince Baseline	-0.164	-0.270 – -0.058	**0.002**	-0.153	-0.291 – -0.014	**0.031**	-0.300	-0.528 – -0.072	**0.010**	0.044	-0.018 – 0.106	0.163
MDT-S	1.472	0.743 – 2.201	**<0.001**									
diagnosis [CU+]	-0.362	-0.708 – 0.016	**0.040**	-0.173	-0.499 – 0.152	0.296	-0.410	-0.820 – -0.001	0.050	-0.071	-0.478 – 0.336	0.732
diagnosis [MCI-]	-1.492	-1.987 – -0.997	**<0.001**	-1.407	-1.873 – -0.941	**<0.001**	-1.447	-1.993 – -0.901	**<0.001**	-1.561	-2.115 – -1.008	**<0.001**
diagnosis [MCI+]	-1.656	-2.086 – -1.227	**<0.001**	-1.468	-1.875 – -1.061	**<0.001**	-1.350	-1.921 – -0.779	**<0.001**	-1.239	-1.900 – -0.577	**<0.001**
age	-0.019	-0.035 – -0.003	**0.021**	-0.015	-0.030 – -0.000	**0.043**	-0.013	-0.032 – 0.007	0.207	-0.033	-0.049 – -0.017	**<0.001**
sex [female]	0.132	-0.150 – 0.414	0.359	0.206	-0.061 – 0.473	0.129	0.060	-0.279 – 0.399	0.763	0.064	-0.254 – 0.383	0.692
Education [years]	0.020	-0.024 – 0.063	0.374	0.023	-0.017 – 0.064	0.256	0.027	-0.023 – 0.077	0.291	0.049	-0.002 – 0.100	0.062
YearsSinceBaseline * MDT-S	0.245	0.016 – 0.473	**0.036**									
MDT-O				2.135	1.397 – 2. 873	**<0.001**						
YearsSinceBaseline * MDT-O				0.165	-0.086 – 0.415	0.196						
ORR-DR							0.121	0.069 – 0 .172	**<0.001**			
YearsSinceBaseline * ORR-DR							0.017	0.002 – 0 .032	**0.027**			
p-tau217										-0.099	-0.249 – 0.050	0.190
YearsSinceBaseline * p-tau217										-0.043	-0.076 – -0.010	**0.010**
Random Effects												
σ^2	0.195			0.191			0.179			0.155		
τ_{00}	0.336 $_{UserID}$			0.268 $_{UserID}$			0.292 $_{UserID}$			0.416 $_{UserID}$		
τ_{11}	0.035 $_{UserID.YearsSinceBaseline}$			0.039 $_{UserID.YearsSinceBaseline}$			0.034 $_{UserID.YearsSinceBaseline}$			0.014 $_{UserID.YearsSinceBaseline}$		
ρ_{01}	0.107 $_{UserID}$			0.223 $_{UserID}$			0.149 $_{UserID}$			0.145 $_{UserID}$		
ICC	0.750			0.754			0.750			0.779		
N	100 $_{UserID}$			100 $_{UserID}$			66 $_{UserID}$			86 $_{UserID}$		
Observations	328			328			215			279		
Marginal R^2 / Conditional R^2	0.550 / 0.888			0.559 / 0.891			0.623 / 0.906			0.494 / 0.888		
AIC	720.829			711.537			479.013			568.680		
